# Granules Harboring Translationally Active mRNAs Provide a Platform for P-Body Formation following Stress

**DOI:** 10.1016/j.celrep.2014.09.040

**Published:** 2014-10-23

**Authors:** Jennifer Lui, Lydia M. Castelli, Mariavittoria Pizzinga, Clare E. Simpson, Nathaniel P. Hoyle, Kathryn L. Bailey, Susan G. Campbell, Mark P. Ashe

**Affiliations:** 1 Faculty of Life Sciences, Michael Smith Building, The University of Manchester, Oxford Road, Manchester M13 9PT, UK; 2 Biosciences Department, Faculty of Health and Wellbeing, Sheffield Hallam University, Howards Street, Sheffield S1 1WB, UK

## Abstract

The localization of mRNA to defined cytoplasmic sites in eukaryotic cells not only allows localized protein production but also determines the fate of mRNAs. For instance, translationally repressed mRNAs localize to P-bodies and stress granules where their decay and storage, respectively, are directed. Here, we find that several mRNAs are localized to granules in unstressed, actively growing cells. These granules play a key role in the stress-dependent formation of P-bodies. Specific glycolytic mRNAs are colocalized in multiple granules per cell, which aggregate during P-body formation. Such aggregation is still observed under conditions or in mutants where P-bodies do not form. In unstressed cells, the mRNA granules appear associated with active translation; this might enable a coregulation of protein expression from the same pathways or complexes. Parallels can be drawn between this coregulation and the advantage of operons in prokaryotic systems.

## Highlights

• Specific mRNAs are localized to cytoplasmic granules in actively growing cells
• After stress, the granules serve as a platform for P-body formation
• Certain glycolytic mRNAs colocalize and are translated in cytoplasmic granules
• Translation of functionally related mRNAs may be coregulated in the granules

## Graphical Abstract

**Figure:**
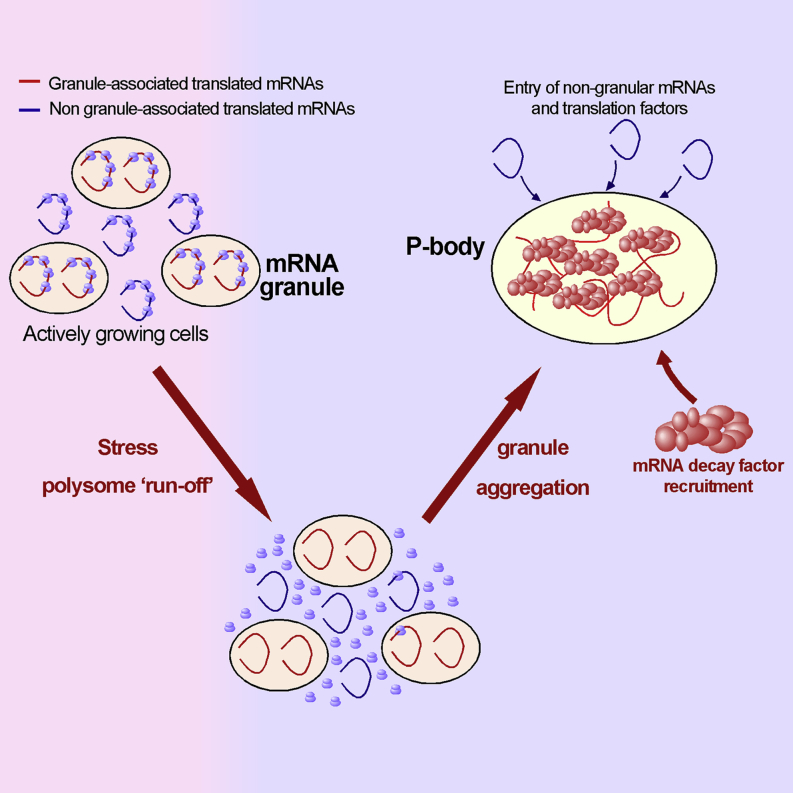

## Teaser

Lui et al. show that specific mRNAs localize to granules in actively growing cells. These mRNA granules are associated with active translation and are proposed to allow coregulation of protein expression from the same pathway or complex. Following stress, the granules aggregate to drive the formation of P-bodies.

## Introduction

A variety of granules or bodies harboring mRNA have been described. In the late 19^th^ century, Franz Nissl identified Nissl bodies in neurons (Schoenberg and Schoenberg, 1979), which were later found to be rich in RNA and ribosomes and associated with endoplasmic reticulum (ER) (Singhrao and Nair-Roberts, 2010). It is now known that mRNAs accumulate on the ER, peroxisomes, and mitochondria, and that translational derepression followed by translocation or import allows coordinated protein production at these sites (Gadir et al., 2011; Schwartz, 2007; Zipor et al., 2009). Integrated control of mRNA localization and translation also provides a more general means to regulate temporal and spatial protein production (Shahbabian and Chartrand, 2012). This is important in many cellular contexts including neuronal dendrites (Kindler and Kreienkamp, 2012), oocytes and early embryos (Lasko, 2009), and even in single-celled eukaryotes, such as the budding yeast, where controls over *ASH1* mRNA localization and translation establish the specificity of mating type switching (Paquin and Chartrand, 2008). Although classically such mechanisms are considered to affect a small number of mRNAs, more recent studies suggest these localization events are much more widespread (Holt and Bullock, 2009).

mRNAs can also become localized under stress conditions to RNA processing bodies (P-bodies) and stress granules, which have roles in mRNA degradation and storage, respectively (Balagopal and Parker, 2009; Buchan and Parker, 2009; Hoyle and Ashe, 2008). Such granules allow translation resumption upon alleviation of the stress and they facilitate adaptation. Importantly, these granules are generally associated with translation repression.

P-bodies were identified by studying the localization of mRNA decay components such as the LSm complex, the Dcp1/Dcp2 decapping complex or the 5′ to 3′ exoribonuclease, Xrn1 (Bashkirov et al., 1997; Ingelfinger et al., 2002; Lykke-Andersen, 2002; van Dijk et al., 2002). An ingenious genetic strategy in yeast showed that mRNA decay can occur in P-bodies (Sheth and Parker, 2003). Since then, a host of RNA binding proteins, translation initiation factors, and mRNA decay factors have been found in P-bodies (Kedersha and Anderson, 2009), as well as miRNAs and components associated with RNA interference (Jackson and Standart, 2007). It has also been shown that P-bodies are induced as a response to certain stresses (Teixeira et al., 2005); for example, glucose starvation causes a rapid and robust inhibition of translation initiation (Ashe et al., 2000), which is followed by P-body formation (Teixeira et al., 2005). Models for the formation of P-bodies after stress posit that the bulk of untranslated mRNA created by the translational inhibition interacts with specific RNA binding proteins and mRNA decay factors to form P-bodies (Franks and Lykke-Andersen, 2008). A key supporting observation is that the inhibition of translation elongation by cycloheximide to prevent mRNA release from the translation machinery impedes P-body formation (Teixeira et al., 2005). In addition, deletions of specific prion-related glutamine/asparagine (Q/N)-rich domains from certain mRNA decay components (e.g., Lsm4p and Edc3p) prevent P-body formation (Decker et al., 2007; Reijns et al., 2008). However, we have recently found that different mRNAs enter P-bodies in separate kinetic waves requiring distinct factors, suggesting that the simple bulk flow model does not explain all mRNA localization to P-bodies (Simpson et al., 2014).

Stress granules also form following translation repression and contain some of the same components as P-bodies (Kedersha and Anderson, 2009). Two unique features of stress granules relative to P-bodies are the absence of certain mRNA decay components, and the presence of the 40S ribosomal subunit (and associated translation initiation factors, e.g., eIF3) (Kedersha and Anderson, 2009). In yeast, two classes of stress granule have been shown to form following translation repression. First, acute stress conditions cause granules similar to mammalian stress granules to form (Buchan et al., 2011; Grousl et al., 2009; Kato et al., 2011). In contrast, glucose starvation leads to the formation of granules harboring some translation initiation factors and RNA binding proteins, but lacking the 40S subunit and eIF3 (Buchan et al., 2008; Hoyle et al., 2007). We have termed this second class of stress granule the EGP-body, after the eIF4*E*, eIF4*G*, and *P*ab1p proteins initially found in the granule. There is a significant lag in the timing of eIF4E, eIF4G, and Pab1p entry into either P-bodies or EGP-bodies after glucose starvation (Hoyle et al., 2007).

In this study, we investigate the localization of mRNAs to granules using live yeast cells. We find that a number of translationally active mRNAs localize to granules even in unstressed cells. This is particularly surprising given that most localization to granules is associated with translation repression. Following glucose starvation, the mRNA granules recruit mRNA decay components to form P-bodies. Certain mRNAs are found in multiple granules per cell, which aggregate following stress. Therefore, P-body formation, and hence the storage or decay of translationally repressed mRNA, is driven by preexisting mRNA granules. Under non-stress conditions, the mRNA granules are not associated with the mRNA decay machinery and active mRNA translation can occur in these granules. This implies that not only can mRNA decay occur in defined bodies within cells, but also mRNA translation can occur in such structures perhaps to allow coregulation of mRNAs involved in similar pathways or complexes.

## Results

### Specific mRNAs Localize to Granules in Exponential Yeast

Studies of the dynamic localization of mRNA in live cells have benefited enormously from the development of strategies tethering GFP to mRNAs using MS2 or other similar RNA binding protein-GFP fusions. In the yeast m-TAG system, MS2 stem loop sequences (MS2L) can be integrated directly and precisely into the genomic copy of any mRNA 3′ UTR avoiding plasmid expression systems. Expression of an inducible MS2 coat protein (CP)/GFP fusion then allows reliable detection of even low-abundance mRNAs (Haim et al., 2007; Zipor et al., 2009). Indeed, such systems can detect single transcripts, and a particular advantage of this system is that, for transcripts bearing multiple MS2 stem loops, no aggregation has been found in the presence CP-GFP fusions (Fusco et al., 2003; Haim et al., 2007; Shav-Tal et al., 2004).

**Figure 1:**
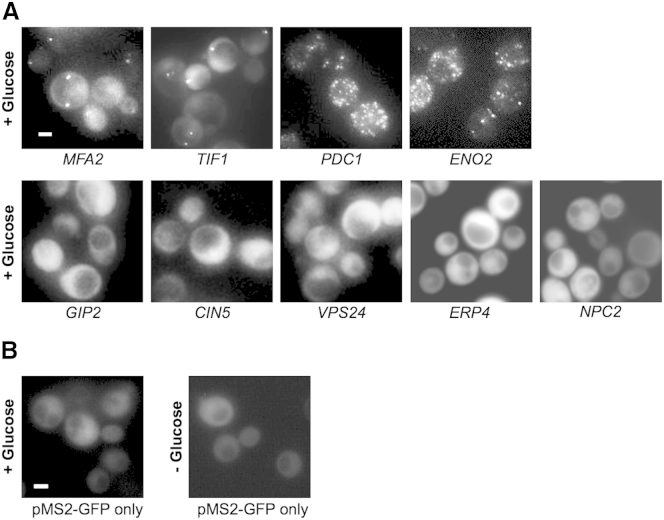
Specific mRNAs Localize into Granules in Unstressed Cells (A) Epifluorescent microscopic z stack images of exponential cells expressing endogenous 3′ UTR MS2L-tagged mRNAs visualized via coexpressed MS2-GFP_3_. (B) Images of controls expressing pMS2-GFP_3_ (ymk1741) in either SCD (+glucose) or SC (−glucose) media. Cells were grown to exponential phase in SCD (+glucose) media prior to imaging. Scale bars, 2 μm. See also Figure S1 and Table S1.

We have previously used the m-TAG system to examine the fate of specific mRNAs to identify two phases in the localization of mRNA to P-bodies (Simpson et al., 2014). We noticed that even in exponentially growing live cells, certain mRNAs were present in granules. For example in this current study, four MS2-tagged mRNAs, *MFA2*, *TIF1*, *PDC1*, and *ENO2* (Figure 1A), were present in granules in exponentially growing cells. It is interesting to note that the *MFA2* and *TIF1* mRNAs were present in one to two granules per cell, whereas *PDC1* and *ENO2* were present in multiple granules per cell (Figure 1A). A similar localization was observed for these mRNAs using MS2-mCherry, which should be less prone to aggregation (Shu et al., 2006), and using Fluorescent in situ hybridization in non-MS2-tagged strains (Figure S1). Five other MS2-tagged mRNAs, *CIN5*, *GIP2*, *VPS24*, *NPC2*, and *ERP4* mRNAs, were not in granules in exponentially growing yeast but were distributed evenly throughout the cytoplasm (Figure 1A). Even though other studies using the m-TAG system have found mRNA in the bud tip or associated with organelles such as the mitochondria or peroxisomes (Gadir et al., 2011; Haim et al., 2007; Zipor et al., 2009), the results for these five mRNAs suggest that many mRNAs exhibit a diffuse cytoplasmic localization. Importantly, as observed previously (Haim et al., 2007), control cells expressing just the pCP-GFP_3_ (lacking MS2L) exhibit weak diffuse GFP signal throughout their cytoplasm, again showing that the MS2-GFP fusion is not prone to aggregation in live cells (Figure 1B).

### mRNA Granules Colocalize with P-Bodies after Stress

**Figure 2:**
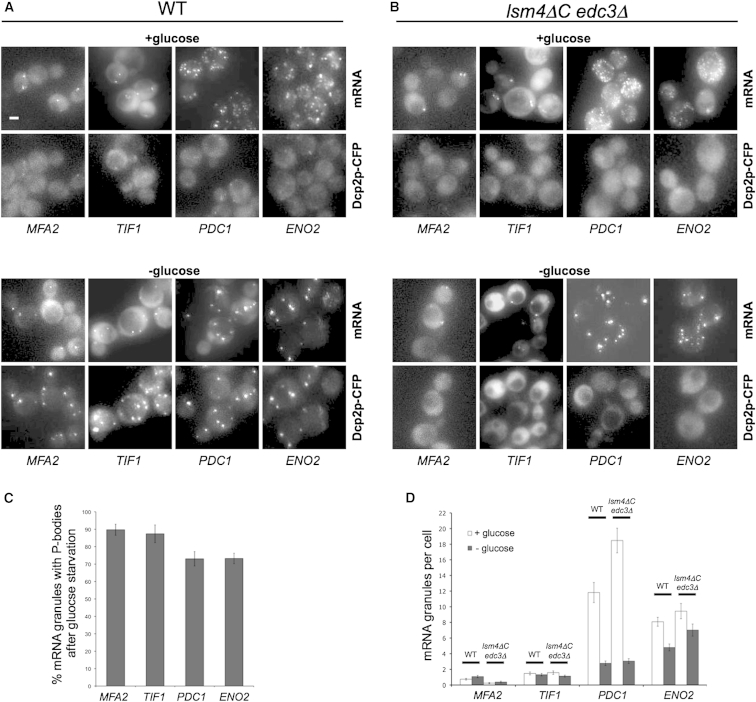
Localization of mRNAs in Granules Does Not Rely on P-Body Formation but Rather Recruits P-Body Components and Coalesce following Glucose Depletion Epifluorescent images of cells grown to exponential phase and then incubated in either SCD (+glucose) or SC media (−glucose) for 10 min. (A) Images of wild-type (WT) cells expressing the P-body marker Dcp2p-CFP as well as MS2L-tagged mRNAs visualized with pMS2-GFP_3_. (B) Images from mutant strains deficient in P-body formation (*edc3Δ lsm4ΔC*) also expressing Dcp2p-CFP, MS2-tagged mRNA, and pMS2-GFP_3_. Scale bar, 2 μm. (C and D) Bar charts showing (C) the percentage of mRNA granules colocalized with P-bodies (Dcp2p) following glucose starvation and (D) the mean number of mRNA granules per cell in wild-type (WT) and *edc3Δ lsm4ΔC* mutants under glucose replete and starvation conditions. z stack merged images were used to count granules across 50 cells. Errors bars are ±SE. See also Figure S2.

P-bodies contain mRNA decay factors and RNA binding proteins and serve as sites for either mRNA degradation or storage following translation repression (Brengues et al., 2005; Sheth and Parker, 2003). Exponentially growing cells expressing the mRNA decapping enzyme, Dcp2p-CFP, as a P-body marker, exhibit little evidence of P-bodies. In contrast, the mRNA granules described above are clearly detected (Figure 2A). We investigated the localization of the *MFA2*, *TIF1*, *PDC1*, and *ENO2* mRNAs following glucose depletion for 10 min, conditions that induce robust P-body formation. Under such conditions, all four mRNAs relocalize to P-bodies (Figures 2A and 2C). Similar observations were made using Dcp1p as a marker for P-bodies (data not shown). Very faint Dcp2p bodies can occasionally be observed in unstressed cells. It seems plausible that these faint occasional granules stem from difficulty in maintaining cells in an unstressed state during the manipulations prior to and during microscopy on the slide. Overall though, in unstressed cells, specific mRNAs are present in granules, and after stress these mRNA-containing granules colocalize with P-body markers.

Edc3p and Lsm4p are both involved in complex mechanisms surrounding mRNA decapping and contain specific protein aggregation domains. Removal of these domains in an *edc3Δ lsm4ΔC* mutant strain causes deficient P-body formation (Decker et al., 2007). To examine whether the mRNA granule localization is reliant upon factors important in P-body formation, we investigated the MS2-tagged mRNAs in *edc3Δ lsm4ΔC* mutant cells. Intriguingly, we found that all four granule-localized mRNAs still localize into granules under conditions where P-body formation is deficient (Figure 2B).

Stress granules called “EGP-bodies” also arise at much later time points following glucose starvation (Brengues and Parker, 2007; Hoyle et al., 2007). It seems highly unlikely that the mRNA granules observed in unstressed cells are the same as EGP-bodies, because EGP-bodies only form after prolonged exposure to cellular stress (Brengues and Parker, 2007; Hoyle et al., 2007; Simpson et al., 2014). Consistent with this, cells expressing eIF4E-RFP, a component of yeast EGP-bodies, did not form granules in unstressed cells; instead, as described previously, eIF4E and other translation factors exhibit robust signal throughout the cytoplasm (Brengues et al., 2005; Campbell et al., 2005; Stage-Zimmermann et al., 2000). Furthermore, colocalization of eIF4E with the mRNA granules was only observed after prolonged periods of glucose starvation in P-bodies (data not shown).

### mRNA Granules Aggregate to Form Fewer More Intense Granules after Glucose Starvation

During the course of these studies, we noticed that the *PDC1* and *ENO2* mRNA-containing granules appear more intense and fewer in number after stress (Figure 2A). Quantification of the number of granules revealed that, for *PDC1* and *ENO2* mRNAs, the granules decrease quite dramatically after glucose starvation (Figure 2D). Interestingly, even though the *edc3Δ lsm4ΔC* mutant strains are deficient in P-body formation, the number of mRNA granules per cell for *PDC1* and *ENO2* still decrease after glucose starvation (Figures 2B and 2D). In addition, neither the localization of mRNA to granules nor the reduction in granule number after glucose starvation relies upon the stress granule assembly protein, Pbp1p (Figure S2).

Therefore, specific mRNAs are in granules under exponential growth conditions and, after glucose starvation, these granules coincide with newly formed P-bodies and are reduced in number. We also examined the change in fluorescent intensity for the granules. For *ENO2* mRNA, there is a 7.6- (±2.1) fold increase in granule intensity after stress and for *PDC1* mRNA there is a 3.9- (±0.7) fold increase. These results suggest that the mRNA granules in unstressed cells coalesce such that fewer, yet more intense granules are present after glucose starvation. These aggregated granules colocalize with newly forming P-bodies, even though P-body formation per se is not a requirement for the coalescence of the mRNA granules.

### Amino Acid Starvation Induces mRNA Granule Coalescence without P-Body Formation

**Figure 3:**
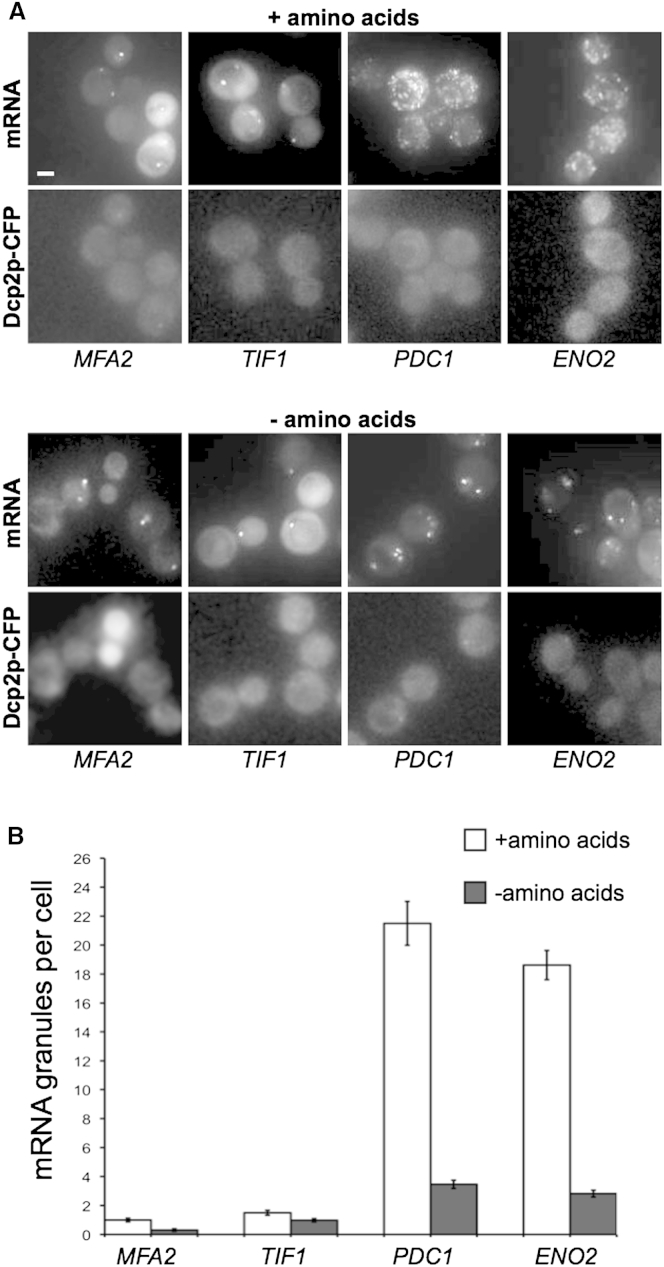
Other Stresses Cause mRNA Granule Aggregation without Inducing P-Bodies Epifluorescent images of cells grown to exponential phase and then incubated in either SCD (+amino acids) or SC-AAs (−amino acids) for 10 min. (A) Images of cells expressing Dcp2p-CFP and MS2L-tagged mRNA/pMS2-GFP_3_. Scale bar, 2 μm. (B) Quantification of the mean number of mRNA granules per cell under unstressed and amino acid starvation conditions. Merged z stacks were used to count the granules from at least 50 cells. Errors bars are ±SE.

Amino acid starvation also inhibits translation initiation via a different pathway from that induced by glucose starvation (Ashe et al., 2000; Castelli et al., 2011). Amino acid starvation targets the guanine nucleotide exchange factor eIF2B via phosphorylation of eIF2α (Wek et al., 2006). This prevents eIF2 recycling to its active GTP bound form and hence inhibits translation initiation (Sonenberg and Hinnebusch, 2009). Furthermore, amino acid starvation does not cause P-body or stress granule formation (Hoyle et al., 2007). Therefore, we followed the mRNA granules that are present in unstressed cells after amino acid starvation. Consistent with previous reports (Hoyle et al., 2007), P-bodies were not observed following amino acid starvation. The *MFA2* and *TIF1* mRNA granules remain unaltered by amino acid starvation, whereas, similar to glucose starvation, the *PDC1* and *ENO2* mRNA granules aggregate following amino acid depletion (Figures 3A and 3B). Therefore, it seems that a general response to the stress-dependent inhibition of translation initiation is the restructuring of specific mRNA granules into larger yet fewer aggregates. The fact that two stresses causing polysome runoff induce this aggregation effect highlights the possibility that polysomes were present in the mRNA granules and that translation was occurring there.

### mRNA Granules Coalesce and Recruit P-Body Components

**Figure 4:**
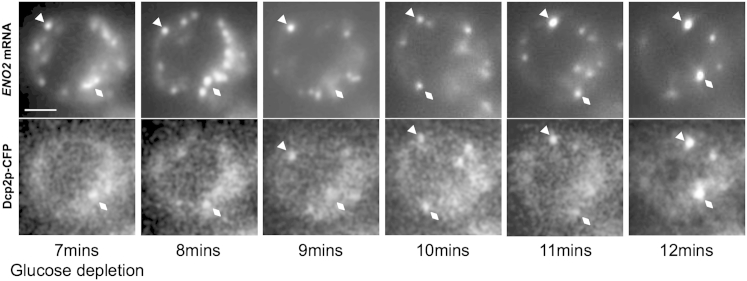
The Recruitment of mRNA Decay Factors to Form P-Bodies Occurs on Preexisting mRNA Granules Epifluorescent images of cells expressing Dcp2p-CFP and MS2L-tagged *ENO2* pMS2-GFP_3_ growing in a microfluidic chamber where the media has been switched for glucose free media and images of cells are collected every minute. The triangle and diamonds highlight mRNA granules, which serve as sites of P-body formation. See also Figure S3.

The experiments above show that specific mRNAs exist in granules, and they suggest that, after the inhibition of translation initiation, *PDC1* and *ENO2* granules fuse to form fewer but larger mRNA granules. Following glucose starvation, mRNA decay components colocalize with these mRNA granules. In order to directly visualize these events in live cells, we used a microfluidic chamber to trap cells while media constantly flows over them. Using this system, yeast can be followed for several divisions, because doubling times are very similar to cells grown under optimal conditions (data not shown). The microfluidic chamber also allows the media flowing over the cells to be exchanged rapidly for glucose free media. We used this system to capture P-body formation and relate this to the mRNA granules that are present prior to glucose starvation. Figure 4 shows one such series of images taken of a cell where P-body formation has been captured. More specifically, Dcp2p is observed accumulating over the course of a few minutes in granules containing *ENO2* mRNA. Highlighted on the images, one granule does not contain Dcp2p 7 min after the switch, but it accumulates over subsequent frames (Figure 4, triangles), whereas a second granule carries very low levels of Dcp2p at the start of the experiment, which intensify over a few minutes (Figure 4, diamonds). What is clear from a number of experiments studying both the *ENO2* and *PDC1* mRNA granules using this system is that the mRNA granules present in the cell prior to starvation become P-bodies by recruiting mRNA decay components (e.g., Figure S3). In addition, these mRNA granules merge to form a smaller number of more intensely fluorescent granules. The coalescence of granules appears to be simultaneous with the recruitment of mRNA decay factors. Therefore, preexisting RNA granules aggregate and nucleate P-body formation following stress.

### Specific mRNA Granules in Unstressed Cells Can Be Associated with Active Translation

In experiments above, the two stresses amino acid and glucose starvation that cause polysome runoff, both induce aggregation of the *ENO2* and *PDC1* mRNA granules. Such an effect suggests that polysomes might have been initially present in the mRNA granules and highlights the possibility that translation might have been occurring there. To shed further light on this possibility, the drug cycloheximide was used to cause a block in translation elongation. Such treatment prevents the formation of P-bodies and stress granules, most likely by trapping mRNA on polysomes (Buchan et al., 2008; Grousl et al., 2009; Kato et al., 2011; Sheth and Parker, 2003). We reasoned that if the mRNA granules observed in unstressed cells are sites of translation, then treatment with cycloheximide should trap ribosome-associated mRNAs in granules preventing their aggregation following stress.

**Figure 5:**
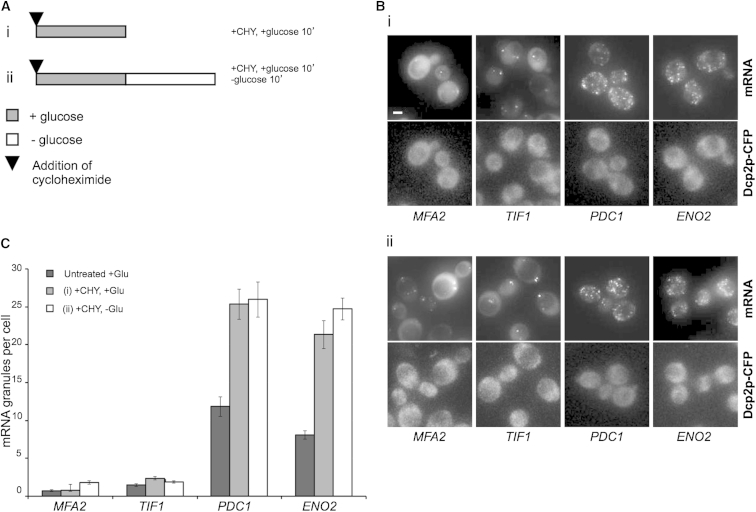
Cycloheximide Treatment Inhibits the Coalescence of mRNA Granules (A) Schematic representing the culture treatment regimen. Gray and white bars represent 10 min growth in media with or without glucose respectively, and arrowheads denote the point of cycloheximide addition. (B) Epifluorescent images of cells expressing Dcp2-CFP and MS2L-mRNA/pMS2-GFP_3_. Cells were grown to exponential phase in media containing glucose and then either (1) treated with cycloheximide for 10 min or (2) treated with cycloheximide for 10 min and then incubated in media lacking glucose for 10 min. (C) Bar chart depicting quantification of the average mRNA granules per cell for the treatments described above. Merged z stacks were used to count the number of granules in 50 cells. Errors bars are ±SE. See also Figure S4.

Therefore, cells were treated with cycloheximide before a brief incubation in media with or without glucose (Figure 5Ai,ii). Little change in the number of *MFA2* and *TIF1* mRNA granules per cell was observed after cycloheximide treatment. Equally, for the *PDC1* and *ENO2* mRNA granules, following cycloheximide, little or no granule aggregation was observed following glucose depletion (Figures 5A and 5B). In fact, for these mRNAs the number of granules per cell increased following cycloheximide treatment regardless of glucose starvation (Figure 5C). The observed increase in mRNA granules in cycloheximide-treated cells could signify an accumulation of ribosome-associated mRNAs in granules, which would support a conclusion that these mRNA granules are sites of translation. This would contrast with a number of other cytoplasmic granules where cycloheximide prevents the flux of mRNA through the granules leading to their disassembly (Andrei et al., 2005; Buchan et al., 2008; Campbell et al., 2005; Grousl et al., 2009; Kato et al., 2011; Kedersha et al., 2000; Teixeira et al., 2005).

**Figure 6:**
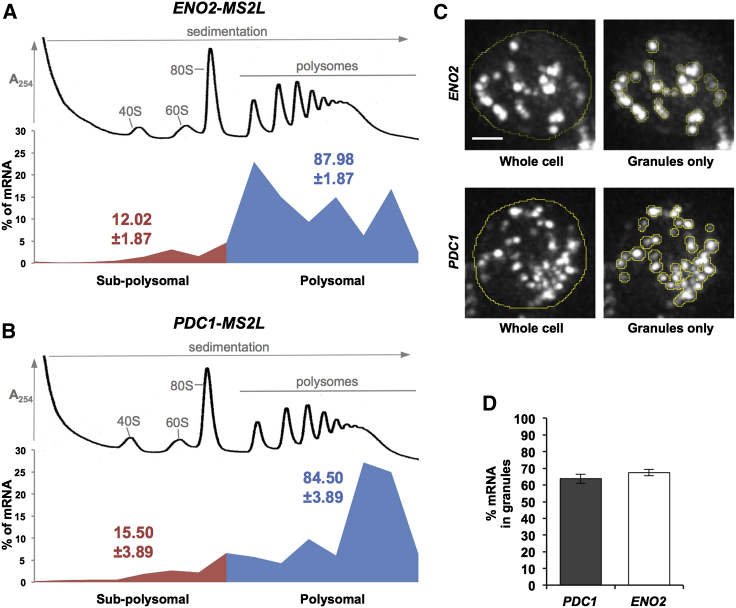
Most *PDC1* and *ENO2* mRNAs Are Associated with Polysomes and Localized to mRNA Granules (A and B) Polysome fractionation and qRT-PCR analysis on RNA prepared from individual fractions across polysome gradients. Polysomes were analyzed as described in Experimental Procedures. Traces depicting the changes in A_254_ across the gradient from the yMK1577 (*ENO2-MS2L*) and yMK1586 (*PDC1-MS2L*) strains grown in YPD are shown. The 40S (small ribosomal subunit), 60S (large ribosomal subunit), 80S (monosome), and polysome peaks are labeled. Below the percentage of each mRNA present in the fractions collected from the polysome gradient is plotted. Blue represents RNA in polysomal regions, whereas red is from the subpolysomal regions of the gradient. The total percentage in polysomal and subpolysomal regions across three repeat experiments is also depicted. (C and D) (C) Representative images depicting the strategy for quantitating the percentage of *PDC1* and *ENO2* mRNAs in granules. Fluorescence was measured for the whole cell and granules only (as defined within the yellow lines), and the percentage of each mRNA present in the granules was calculated and plotted in (D). Scale bar, 1 μm. Errors bars are ±SE. See also Figure S4.

In order to further assess the possibility that translation is occurring in these granules, we carefully quantitated the level of the *ENO2* and *PDC1* mRNAs that are found associated with polysomes so that it could be compared to the level found in granules. Figure 6A shows that, consistent with other estimates of translation efficiency for these mRNAs (Arava et al., 2005; Brar et al., 2012), greater than 80% of each mRNA is present in the polysome fractions of gradients. Given this and previous results, it seems unlikely that the mRNAs accumulate on polysomes due to ribosomal stalling. However, this was further tested using a polysomal runoff analysis that has been previously used to assess the level of translational elongation (Anand et al., 2003; Shenton et al., 2006). Here, induction of polysome runoff by the inhibition of translation initiation (Ashe et al., 2000) led to a dramatic reduction in the level of *PDC1* and *ENO2* in the polysomal fractions (Figure S4). This suggests that the ribosomes on these mRNAs are fully capable of active elongation and hence are not stalled.

In order to measure the proportion of each mRNA in the granules, cells from the same cultures used for the polysome analysis were prepared for fluorescent microscopy. mRNA levels in the granules were estimated by deconvoluting a Z-series of images, as depicted in Figure 6B and measuring the fluorescent intensity in the granules as a proportion of the total fluorescent intensity present in the cell. This analysis suggests that ∼65%–70% of the GFP fluorescence is present in granules. It is difficult to explain how such a large proportion of the two mRNAs can be present in the mRNA granules and also present on polysomes without the polysome bound mRNAs being present in granules and hence translated there.

### Colocalization and Coordinated Granular Translation of the *PDC1* and *ENO2* mRNAs

**Figure 7:**
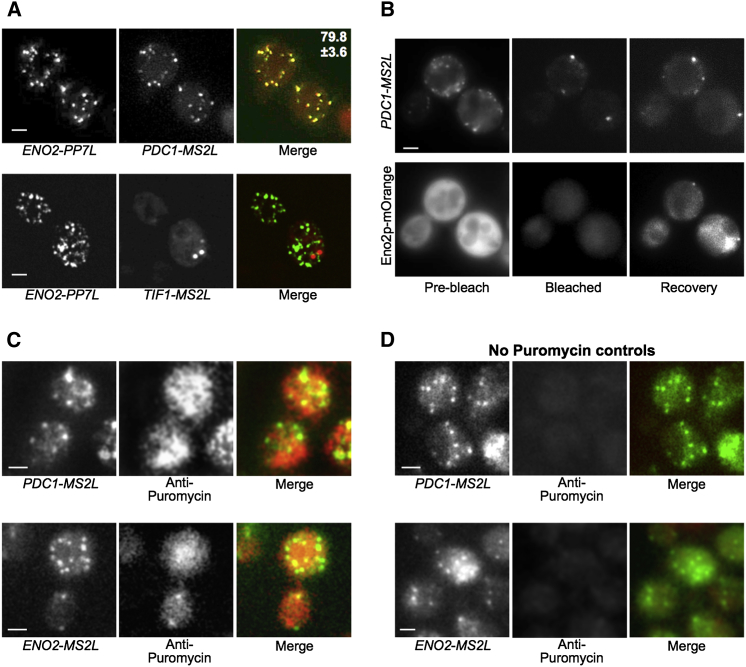
*PDC1* and *ENO2* mRNAs Are Translated in the Same mRNA Granules (A) Epifluorescent microscopic z stack images of exponential cells expressing endogenous PP7L-tagged *ENO2* (visualized via PP7-GFP_2_, left images) and either MS2L-tagged *PDC1* or MS2L-tagged *TIF1* (visualized via coexpressed MS2-mCherry_3_, middle images). Merged images are shown (right) with the percentage GFP granules overlapping with mCherry granules quantified across 50 cells for *ENO2* v *PDC1*. No colocalization was observed for *ENO2* v *TIF1*. Error bar is ±SE. (B) Figure shows a FRAP experiment on the Eno2p-mOrange bearing strain yMK1993 where recovery after photobleaching is followed relative to the localization of the *PDC1-MS2L* mRNA (visualized using MS2-GFP_3_). Prebleach, bleached, and recovery images are shown for *PDC1* mRNA (top row) and mOrange-tagged Eno2p protein (bottom row). (C) Immunofluoresence using an antipuromycin antibody on cells treated with puromycin/cycloheximide to trap puromycin at the site of protein synthesis (center panels). The MS2-GFP_3_ mRNA signal for *PDC1* and *ENO2* is maintained during the procedure (left panels). Merged images show the overlap of the puromycin signal with the mRNA granules (right panels). (D) As in (C), except puromycin was omitted from the procedure. Scale bars, 2 μm throughout. See also Figures S5 and S6.

Both the *PDC1* and the *ENO2* mRNAs are involved in the glycolytic fermentation of glucose to ethanol in yeast. One intriguing possibility is that the localization of these mRNAs to granules in exponentially growing cells could facilitate a coordinated production of proteins from the same metabolic pathway. A prediction of this would be that these mRNAs should largely colocalize to the same granules within the cell. To test this in live cells, coordinate use was made of the MS2 and the PP7 systems: both phage RNA binding protein strategies have been previously combined to study the localization of different mRNAs in the same cells (Hocine et al., 2013). A strain was constructed bearing *PDC1* tagged with MS2 stem loops and *ENO2* tagged with PP7 stem loops. MS2-mCherry3 and PP7-GFP fusion proteins were coexpressed in this strain. Figure 7A shows that the *ENO2* and *PDC1* mRNAs largely colocalize into the same mRNA granules. A conservative estimate of this given the differences in background fluorescence of mCherry relative to GFP is that 70% of granules colocalize (Figure 7A). In contrast, no colocalization was observed for a strain where *ENO2* and *TIF1* mRNAs were evaluated using the same approach (Figure 7A).

The fact that the pattern of localization for the *PDC1* and *ENO2* mRNAs overlapped allowed us to devise a strategy to study the localization of the protein product for one of these mRNAs relative to the localization of the mRNA for the other. Therefore, we generated a strain where the endogenous *ENO2* gene coding sequence was fused to Orange Fluorescent protein (OFP). This strain also carried the MS2-tagged *PDC1* gene. Fluorescent recovery after photobleaching (FRAP) experiments were performed using this strain, where the OFP was specifically photobleached and the production of new unbleached protein could then be followed relative to the localization of the *PDC1* mRNA. In Figure 7B, prior to photobleaching the mRNA is present in granules, whereas the Eno2p-OFP protein is distributed relatively evenly throughout the cytoplasm, as has previously been found for this and other glycolytic enzymes (Tkach et al., 2012). Immediately following photobleaching, the mRNA is still visible in granules but the Eno2p-OFP signal is dramatically reduced, although problems with autofluorescence mean the signal never entirely disappears. Following a 10 min recovery period, the accumulation of newly fluorescent Eno2p-OFP, which is dependent on new protein synthesis (Figure S5), was observed at precisely the same loci as the most intense *PDC1* mRNA granules (Figure 7B). Indeed, across a number of different experiments whenever protein was observed to appear in granules this overlapped with an intense mRNA granule. Although suggestive, these data for the glycolytic mRNAs do not prove that their translation occurs in cytoplasmic granules, because the OFP fluorescent molecule will take time to fold and there may be phototoxic effects associated with the photobleaching.

On the basis of these caveats, we undertook a further assessment of translation using a ribopuromycilation method (David et al., 2011) adapted to yeast. Puromycin labeling has been used in a number of studies to label the sites of protein synthesis (Schmidt et al., 2009; Willett et al., 2011; David et al., 2012). The difficulty in yeast has always been getting the puromycin into yeast cells under conditions where they are still actively translating. Here, we used a lyticase treatment step, which we showed, using polysome analysis, does not impact upon global protein synthesis (Figure S6A) but still allows sufficient puromycin into the cell to label proteins (Figure S6B). Addition of cycloheximide to cells prevents puromycin mediated runoff and hence maintains polysomes (Figure S6A). Using these conditions, we performed immunofluorescence using an antibody specific to puromycin. Even though the cells have been treated for immunofluorescence, the MS2-GFP system still allows the identification of *PDC1* and *ENO2* mRNA granules (Figures 7C and 7D). The analysis further shows that puromycin accumulates throughout the yeast cytoplasm, as might be expected for cells that are very actively translating their mRNA content. Furthermore, a proportion of the puromycin-marked sites of protein synthesis overlap with both the *PDC1* and *ENO2* mRNA granules (Figure 7C). Even though some mRNA granules localize less well with the puromycin signal than others and hence we cannot formally rule out that some of the mRNA granules contain translationally repressed mRNAs, these data do further enhance the conclusion that mRNA translation can occur in mRNA granules containing the *PDC1* and *ENO2* mRNAs.

## Discussion

In this study, we describe the localization of various mRNAs using the m-TAG system (Haim et al., 2007). Surprisingly, we show that certain mRNAs exist in granules in unstressed cells and that these granules serve as sites of translation. Glucose starvation leads to the rapid inhibition of translation and formation of P-bodies (Ashe et al., 2000; Teixeira et al., 2005). Under such conditions, the mRNA granules merge to form larger granules that serve as a platform for the recruitment of mRNA decay factors during the formation of P-bodies.

A key finding in this study is the localization of specific mRNAs to granules in unstressed cells, and an important question relates to the function of these granules. Several lines of evidence suggest that mRNA translation occurs in such granules. First, in contrast to granules bearing translationally repressed mRNAs, such as P-bodies and stress granules, where cycloheximide inhibits formation by trapping mRNAs in polysomes, the mRNA granules observed in unstressed cells are either unaffected or increase in number following cycloheximide treatment. Since for some mRNAs cycloheximide causes a rapid increase in the quantity of mRNA granules per cell, and it targets elongating ribosomes, this suggests that elongating ribosomes are present in the granules. Second, two different conditions known to induce polysome runoff, glucose and amino acid starvation, both lead to a decrease in the number of such mRNA granules per cell via aggregation. As polysome runoff induces this aggregation, it seems highly likely that polysomes are present in the granules prestress. Third, the mRNAs investigated are highly expressed in unstressed conditions with a large proportion of the mRNA being polysome associated: equally a large proportion of each mRNA is localized to granules in unstressed cells, suggesting that much of the mRNA present in the granules is being translated. In addition, a FRAP strategy to follow newly made protein relative to the mRNA granules reveals the accumulation of protein in the granules. Finally, a substantial proportion of the mRNA granules overlap with sites labeled using a puromycin assay. We believe this provides compelling evidence that translation can occur in the mRNA granules that we have identified.

mRNA-containing granules have been widely described as part of the cellular response to stress and accumulate as a direct consequence of translation repression (Balagopal and Parker, 2009; Kedersha and Anderson, 2009). They are not associated with localized protein production; rather, they are thought to play roles in mRNA decay (P-bodies) and storage (stress granules); though the boundaries between these functions are somewhat blurred, a number of mRNAs present in P-bodies can re-enter the translated pool following adaptation to stress, and some mRNA decay components are present in stress granules (Arribere et al., 2011; Brengues et al., 2005; Kedersha and Anderson, 2009). Recent work suggests that RNA and RNA binding protein aggregation in cell-free systems rely upon low-complexity protein domains (Han et al., 2012; Kato et al., 2012). Two such low-complexity Q/N-rich domains in the Lsm4p and Edc3p proteins are critical in the formation of P-bodies (Decker et al., 2007; Reijns et al., 2008). Here, we show that aggregation of the mRNA granules after stress still occurs in an *edc3Δ lsm4ΔC* mutant, even though the recruitment of mRNA decay factors and formation of P-bodies is precluded. Therefore, it appears that the mRNA granules serve as precursors to P-bodies and that mRNA granule aggregation forms part of P-body formation. The question remains what causes this aggregation of mRNA granules after the polysome runoff caused by either glucose or amino acid starvation. One possibility is that the lack of ribosomes gives access to other RNA binding proteins with similar low-complexity domains, and these mediate aggregation of the granules to nucleate P-body formation, the latter occurring only under glucose starvation conditions and not amino acid starvation. A possible explanation for the stress specificity in P-body formation lies in recent studies highlighting the connection between the cAMP-dependent protein kinase (PKA) pathway and P-bodies (Ramachandran et al., 2011; Tudisca et al., 2010, 2012). For instance, given the well-established connections between glucose signaling and the PKA pathway, it is entirely plausible that signaling inputs from the PKA pathway promote the recruitment of mRNA decay factors to the aggregated mRNA granules under glucose starvation conditions, but these are not activated under amino acid starvation conditions.

As highlighted above, in this study we describe mRNA-containing granules in unstressed cells where, even though the mRNA is likely translated, there does not appear to be any specific requirement for the localization of the resulting protein product, nor does the mRNA show a localization pattern indicative of ER, peroxisomal, or mitochondrial localization. The specific mRNAs that are localized produce proteins involved in processes such as glycolysis and translation. These proteins, eIF4A, Eno2p, and Pdc1p, have a broad cytoplasmic localization (Campbell et al., 2005; Huh et al., 2003). Furthermore, we find two classes of granule, with either 1–2 or 10–20 granules per cell; the more abundant class includes mRNAs encoding enzymes involved in the glycolytic pathway. Therefore, what is the function of these mRNA granules?

One possibility is that the granules promote high-efficiency translation: if so, why isn’t any mRNA encoding an abundant protein present in such RNA granules? For instance, here we describe five highly expressed mRNAs that are not present in mRNA granules. Another possibility relates to the response to stress: perhaps the primary function of these granules is to promote P-body formation? However, mutants in P-body formation show no deficiencies in translational repression or mRNA decay (Decker et al., 2007). Therefore, our favored hypothesis is that the granules facilitate coordinated protein production, maybe allowing a precise stoichiometric balance in protein levels across specific pathways or within large multimeric complexes. It is entirely possible that these mRNAs colocalize into granules due to cotranslational folding and interaction at the level of the protein nascent chains allowing efficient assembly of proteins into complexes. Such a model allows parallels to be drawn with prokaryotic systems where mRNAs are produced as operons allowing the coordinated synthesis of functionally related proteins. Intriguingly, the two mRNAs that we find in 10 to 20 granules per cell are both involved in glucose fermentation. Coordination of glycolysis via interaction between particular enzymes is well established (Campanella et al., 2005). Indeed, in yeast from the comprehensive atlas of protein-protein interactions (Collins et al., 2007), Eno2p (Enolase) interacts with Fba1p (Aldolase), Pdc1p (Pyruvate Decarboxylase), and Pgk1p (Phosphoglycerate kinase). Such metabolic coordination could start at the level of mRNA localization where it is plausible that translation of colocalized mRNAs facilitates interactions due to the proximity of the synthesis and subsequent folding pathways.

## Experimental Procedures

### Strains and Plasmids

Strains used in this study are listed in Table S2. Proteins were C-terminally tagged and verified by PCR (Campbell et al., 2005). MS2 binding sites (MS2L) were inserted into the 3′ UTR of genes and were verified using PCR and RT-PCR. MS2 tagging reagents were kindly provided by Jeff Gerst (Haim et al., 2007). PP7 binding sites (PP7L) were inserted into the 3′ UTR using a similar strategy (Hocine et al., 2013). PP7 tagging reagents were purchased from Addgene. The *edc3Δ lsm4ΔC* mutant (kindly provided by J. Hasek [Grousl et al., 2009]) was backcrossed four times to W303-1A and then backcrossed to the MS2L-tagged strains to generate *edc3Δ lsm4ΔC DCP2-CFP* MS2L-mRNA strains.

### Growth Conditions

Cells were grown at 30°C to OD_600_ 0.5 in synthetic complete medium with 2% glucose (SCD) (Sherman, 1991). Cells were incubated for 1 hr in SCD media lacking methionine to induce expression of pCP-GFP_3_. For stress conditions, cells were incubated in media lacking glucose (SC) or lacking amino acids (SC-AA) for 5 or 10 min as indicated. Where indicated, lyticase treatment (1 mg/ml) was conducted in SCD media with 1 M sorbitol for 1 hr, puromycin was added at a final concentration of 1 mg/ml, and cycloheximide was added at a final concentration of 100 μg/ml.

### Microscopy and Quantification

Epifluorescent images from a Delta Vision (Applied Precision) microscope using a 100×/1.40 numerical aperture oil plan Apo objective were collected using a Coolsnap HQ (Photometrics) camera with Softworx 1.1 software (Applied Precision) at a Z-spacing of 500 nm. Optical Z-sections were processed with ImageJ (NIH) using deconvolution. Representative cells are shown from experiments repeated at least three times. Granules per cell were counted using 50 cells for each mRNA in triplicate. Time-course experiments were performed using a microfluidic flow device and a Y04C plate (CellASIC) with 5 psi leading to a chamber refresh every minute. Cells were added to the inlet well, and 300 μl of SCD and SC media was added to solution wells. SCD media was switched to SC, and images were collected at 1 min intervals. For quantitation of the percentage mRNA in granules, the intensity of fluorescence was measured using the ImageJ software package for at least 20 cells. The corrected total fluorescent intensity for the whole cell and for the granules was measured to calculate the percentage of fluorescence in granules. For the photobleaching experiment, a 490/20 nm filter was used and cells were bleached for 10 min before recovery after photobleaching was monitored. Colocalization of PP7- and MS2-tagged mRNAs was assessed by scoring granules across 50 cells, and the average percentage colocalization was calculated.

### Polysome Fractionation and Quantitative RT-PCR

Polysome fractionation and RNA preparation were carried out as previous (Castelli et al., 2011) with the following modifications. Fifteen fractions were collected across the gradient into two volumes Trizol (Life Technologies). Four nanograms luciferase control RNA (Promega) was spiked into each fraction, and then the RNA was extracted, precipitated, and resuspended in diethyl-pyrocarbonate-treated water. The RNA was converted to cDNA using a Protoscript M-MuLV Taq RT-PCR kit (New England Biolabs), and quantitative RT-PCR (qRT-PCR) was performed with the CFx Connect Real-Time system with iTaq Universal SYBR Green Supermix (Bio-Rad). Samples were run in triplicate and normalized to luciferase RNA, and the fold change was calculated using 2^−ΔCt^ for each tested RNA. From this, the percentage of the test RNA in each fraction was calculated.

### Fluorescent In Situ Hybridization

DIG-UTP labeled RNA probes were generated using primers with T3 and T7 promoter ends and a MAXIscipt In Vitro transcription kit (Life Technologies). Probes were hydrolyzed using sodium bicarbonate/carbonate solution. Fluorescent in situ hybridization was carried out as previously described (Youk et al., 2010) with the following modifications. Samples were incubated with a sheep antidigoxigenin antibody (1:400, Roche) following incubation with RNA probes and then incubated with a donkey anti-sheep Alexa Fluor 555 antibody (Invitrogen). Samples were counterstained with DAPI (Invitrogen) and then mounted to a microscope slide using ProLong Gold Antifade reagent (Life Technologies).

### Immunofluorescence

Immunoflourescence was performed as described previously (Campbell et al., 2005) with the following modifications. Sorbitol (1 M) was included in all growth media. During the induction of pCP-GFP_3_, 1 mg/ml lyticase was added. Puromycin and cycloheximide treatments were carried out prior to fixation with 3.7% formaldehyde. Finally, the antipuromycin monoclonal antibody 12D10 (Millipore) and goat anti-mouse Texas-red-conjugated secondary antibody (Abcam) were used according to the manufacturer’s guidelines.

## Supplemental Information

Document S1. Figures S1–S6 and Tables S1 and S2

Document S2. Article plus Supplemental Information
