# Supplementary material for: Granules Harboring Translationally Active mRNAs Provide a Platform for P-Body Formation following Stress
Source: Cell Rep. 2014 Oct 23;9(3):944–54. doi: 10.1016/j.celrep.2014.09.040 (PMC4536303; doi:10.1016/j.celrep.2014.09.040)
Supplement: Document S1. Figures S1–S6 and Tables S1 and S2 [file mmc1.pdf]

Table S1 List of selected genes and their functions. Related to Figure 1.

**Gene**	**Protein**	**Function**
ENO2	Enolase II	Enzyme involved in conversion of 2-phosphoglycerate to phosphoenolpyruvate during glycolysis and gluconeogenesis
MFA2	Mating Factor A	Mating factor A, expressed by a-cells and interacts with alpha-cells to induce cell cycle arrest and other responses leading to mating.
PDC1	Pyruvate Decarboxylase	Enzyme decarboxylates pyruvate to acetaldehyde during alcoholic fermentation.
TIF1	Translation Initiation Factor eIF4A	DEAD box RNA helicase that binds to eIF4G during translation intiation and couples ATPase activity to RNA binding and unwinding.
GIP1	Glc7-Interacting Protein	Meiosis-specific regulatory subunit of the Glc7p protein phosphatase, regulates spore wall formation and septin organization, required for expression of some late meiotic genes and for normal localization of Glc7p
CIN5	Chromosome Instability	Basic leucine zipper (bZIP) transcription factor of the yAP-1 family, physically interacts with the Tup1-Cyc8 complex and recruits Tup1p to its targets, mediates pleiotropic drug resistance and salt tolerance, nuclearly localized under oxidative stress and sequestered in the cytoplasm by Lot6p under reducing conditions
VPS24	Vacuolar Protein Sorting	One of four subunits of the endosomal sorting complex required for transport III (ESCRT-III), forms an ESCRT-III subcomplex with Did4p, involved in the sorting of transmembrane proteins into the multivesicular body (MVB) pathway
NPC2	Niemann Pick type C homolog	Functional homolog of human NPC2/He1, which is a cholesterol-binding protein whose deficiency causes Niemann-Pick type C2 disease involving retention of cholesterol in lysosomes
ERP4	Emp24p/Erv25p Related Protein	Protein with similarity to Emp24p and Erv25p, member of the p24 family involved in ER to Golgi transport

Table S2. Yeast strains used in this study. Related to Experimental procedures.

Strain Name	Genotype	Source
yMK466	MATa ADE2 his3-11,15 leu2-3,112 trp1-1 ura3-1	Ashe strain collection
yMK467	MATα ADE2 his3-11,15 leu2-3,112 trp1-1 ura3-1	Ashe strain collection
yMK1307	yMK466 CDC33-RFP::NAT	Ashe strain collection
yMK1514	yMK466 ENO2-MS2L	This study
yMK1577	yMK466 ENO2-MS2L p[MS2-GFP_3 HIS3 CEN]	This study
yMK1586	yMK467 PDC1-MS2L p[MS2-GFP_3 HIS3 CEN]	This study
yMK1587	yMK467 ENO2-MS2L p[MS2-mCh_3 HIS3 CEN]	This study
yMK1588	yMK467 TIF1-MS2L p[MS2-mCh_3 HIS3 CEN]	This study
yMK1589	yMK467 PDC1-MS2L p[MS2-mCh_3 HIS3 CEN]	This study
yMK1606	MATa leu2 ura3 his3 lys2 met15 RPG1-RFP::G418 NGR1-GFP::HIS3 lsm4ΔC::LEU2 edc3::URA3	Jiri Hasek (CRY1041)
yMK1628	yMK467 DCP2-CFP::TRP1	This study
yMK1629	yMK467 CDC33-RFP::NAT pbp1::TRP1 PDC1-MS2L p[MS2-GFP_3 HIS3 CEN]	This study
yMK1631	yMK467 CDC33-RFP::NAT pbp1::TRP1 TIF1-MS2L p[MS2-GFP_3 HIS3 CEN]	This study
yMK1635	yMK467 MFA2-MS2L p[MS2-mCh_3 HIS3 CEN]	This study
yMK1637	yMK467 CDC33-RFP::NAT pbp1::TRP1 MFA2-MS2L p[MS2-GFP_3 HIS3 CEN]	This study
yMK1639	yMK467 CDC33-RFP::NAT pbp1::TRP1 ENO2-MS2L p[MS2-GFP_3 HIS3 CEN]	This study
yMK1645	yMK467 CDC33-RFP::NAT DCP2-CFP::TRP1 MFA2-MS2L p[MS2-GFP_3 HIS3 CEN]	This study
yMK1646	yMK467 CDC33-RFP::NAT DCP2-CFP::TRP1 PDC1-MS2L p[MS2-GFP_3 HIS3 CEN]	This study
yMK1647	yMK467 CDC33-RFP::NAT DCP2-CFP::TRP ENO2-MS2L p[MS2-GFP_3 HIS3 CEN]	This study
yMK1648	yMK467 CDC33-RFP::NAT DCP2-CFP::TRP1 TIF1-MS2L p[MS2-GFP_3 HIS3 CEN]	This study
yMK1725	yMK467 lsm4ΔC::LEU2 edc3::URA3	This study
yMK1726	yMK466 lsm4ΔC::LEU2 edc3::URA3	This study
yMK1727	yMK466 lsm4ΔC::LEU2 edc3::URA3 CDC33-RFP::NAT DCP2-CFP::TRP1 TIF1-MS2L p[MS2-GFP_3 HIS3 CEN]	This study
yMK1728	yMK466 lsm4ΔC::LEU2 edc3::URA3 CDC33-RFP::NAT DCP2-CFP::TRP1 ENO2-MS2L p[MS2-GFP_3 HIS3 CEN]	This study
yMK1729	yMK466 lsm4ΔC::LEU2 edc3::URA3 CDC33-RFP::NAT DCP2-CFP::TRP1 PDC1-MS2L p[MS2-GFP_3 HIS3 CEN]	This study
yMK1730	yMK466 lsm4ΔC::LEU2 edc3::URA3 CDC33-RFP::NAT DCP2-CFP::TRP1 MFA2-MS2L p[MS2-GFP_3 HIS3 CEN]	This study
yMK1741	yMK467 p[MS2-GFP_3 HIS3 CEN]	This study
yMK1819	yMK466 dhh1::G418 CDC33-RFP::NAT DCP2-CFP::TRP1 MFA2-MS2L p[MS2-GFP_3 HIS3 CEN]	This study
yMK1820	yMK466 dhh1::G418 CDC33-RFP::NAT DCP2-CFP::TRP1 PDC1-MS2L p[MS2-GFP_3 HIS3 CEN]	This study
yMK1821	yMK466 dhh1::G418 CDC33-RFP::NAT DCP2-CFP::TRP1 ENO2-MS2L p[MS2-GFP_3 HIS3 CEN]	This study
yMK1822	yMK466 dhh1::G418 CDC33-RFP::NAT DCP2-CFP::TRP1 TIF1-MS2L p[MS2-GFP_3 HIS3 CEN]	This study
yMK1830	yMK466 CDC33-RFP::NAT DCP2-CFP::TRP1 GIP2-MS2L p[MS2-GFP_3 HIS3 CEN]	This study
yMK1831	yMK466 CDC33-RFP::NAT DCP2-CFP::TRP1 CIN5-MS2L p[MS2-GFP_3 HIS3 CEN]	This study
yMK1832	yMK466 CDC33-RFP::NAT DCP2-CFP::TRP1 VPS24-MS2L p[MS2-GFP_3 HIS3 CEN]	This study
yMK1833	yMK466 CDC33-RFP::NAT DCP2-CFP::TRP1 NPC2-MS2L p[MS2-GFP_3 HIS3 CEN]	This study
yMK1834	yMK466 CDC33-RFP::NAT DCP2-CFP::TRP1 ERP4-MS2L p[MS2-GFP_3 HIS3 CEN]	This study
yMK1993	yMK467 ENO2-OFP::HPH PDC1-MS2L p[MS2-GFP_3 HIS3 CEN]	This study
yMK2257	yMK467 ENO2-PP7L PDC1MS2L p[MS2 -mCh_3 HIS3 CEN] p[PP7 -GFP_3 URA3]	This study
yMK2262	yMK467 ENO2-PP7L TIF1MS2L p[MS2 -mCh_3 HIS3 CEN] p[PP7 -GFP_3 URA3]	This study

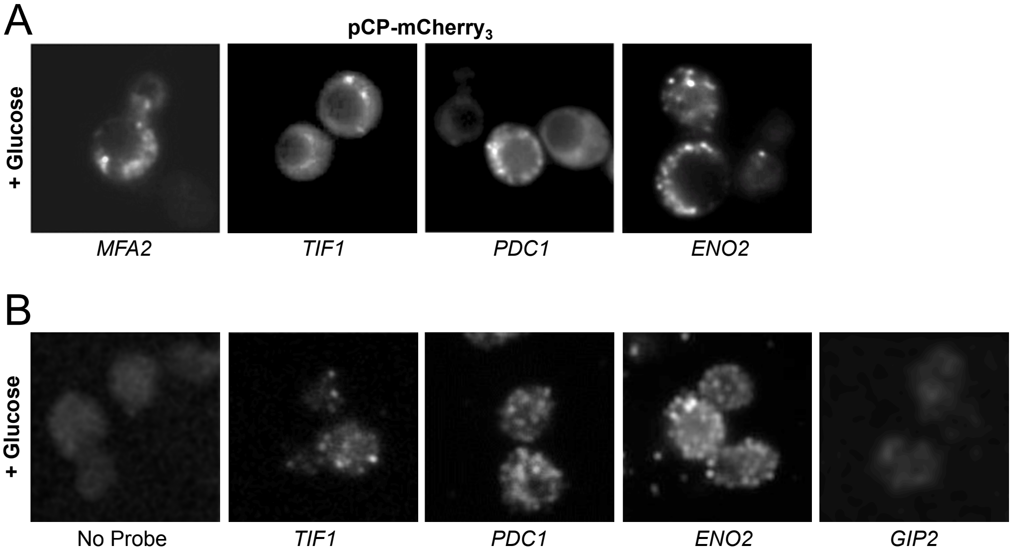

Figure S1. Related to Figure 1. (A) The localization of mRNA using pMS2-mCherry$_3$ in strains bearing MS2-tagged mRNAs (B) Fluorescence *in situ* hybridisation on W303-1A wild type strains using the indicated probes.

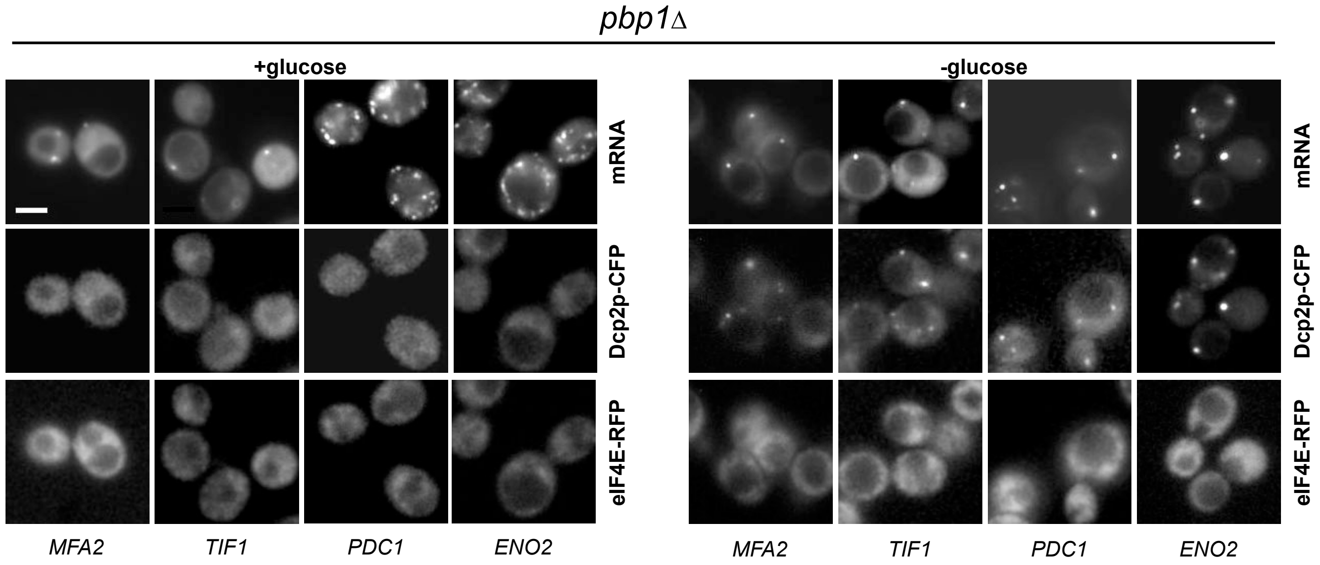

Figure S2. Related to Figure 2. mRNA aggregation is not dependent on EGP-body assembly. Epifluorescent images of mutant strains deficient in EGP-body assembly (*pbp1Δ*) expressing Dcp2p-CFP, eIF4E-RFP, MS2 tagged mRNA and pMS2-GFP_3. Cells were grown to exponential phase and then incubated in either SCD (+glucose) or SC (-glucose) media for 10minutes.

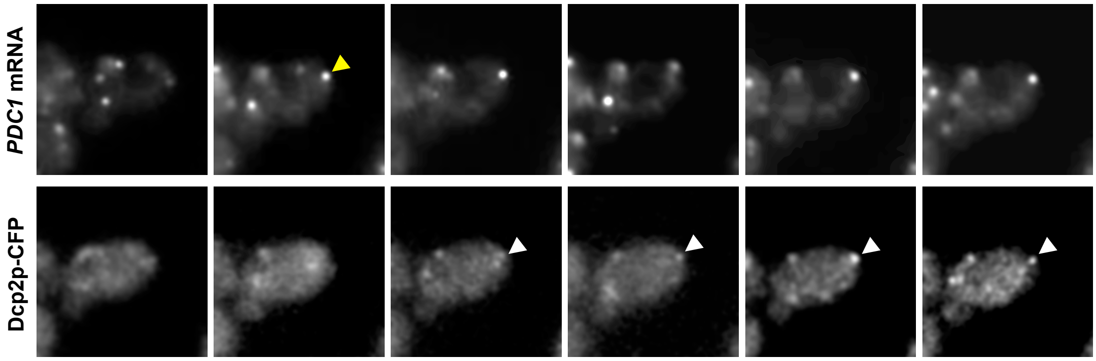

Figure S3. Related to Figure 4. The recruitment of mRNA decay factors to form P-bodies occurs on pre-existing *PDC1* mRNA containing granules. Epifluorescent images of cells expressing Dcp2p-CFP and MS2L-tagged *PDC1* pMS2-GFP$_3$ growing in a microfluidic chamber where the media has been switched for glucose free media and images of cells are collected every minute. Six consecutive images are shown at the point where P-bodies form. The yellow triangle denotes the aggregation of *PDC1* mRNA granules and the white triangles highlight the gradual recruitment of Dcp2p to this granule.

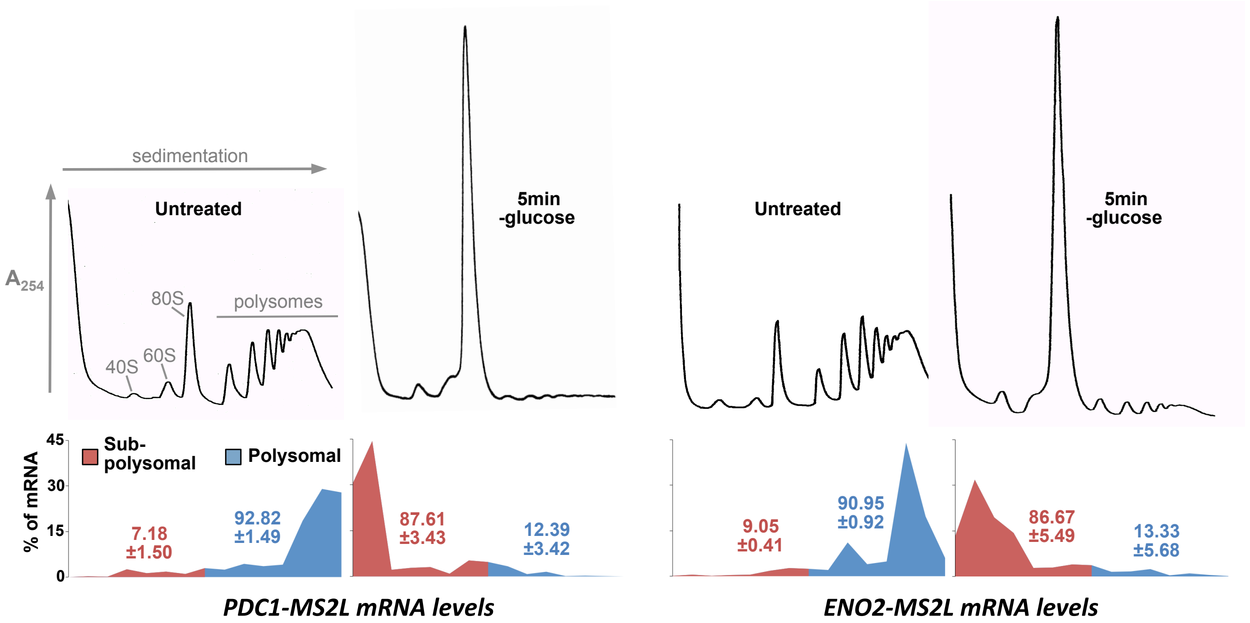

Figure S4. Related to Figure 6. *ENO2* and *PDC1* mRNAs are largely polysome associated and the ribosomes can efficiently run-off these mRNAs. Polysome fractionation and qRT-PCR analysis on RNA prepared from individual fractions across polysome gradients. Traces are shown depicting the changes in A_{254} across the gradient from the yMK1577 (*ENO2-MS2L*) and yMK1586 (*PDC1-MS2L*) strains either untreated or following 5min without glucose to induce rapid ribosome run-off. The 40S (small ribosomal subunit), 60S (large ribosomal subunit), 80S (monosome) and polysome peaks are labelled. Below the percentage of each mRNA present in the fractions collected from the polysome gradient is plotted. Blue represents RNA in polysomal regions whereas red is from the sub-polysomal regions of the gradient. The total percentage in polysomal and sub-polysomal regions across three repeat experiments is also depicted.

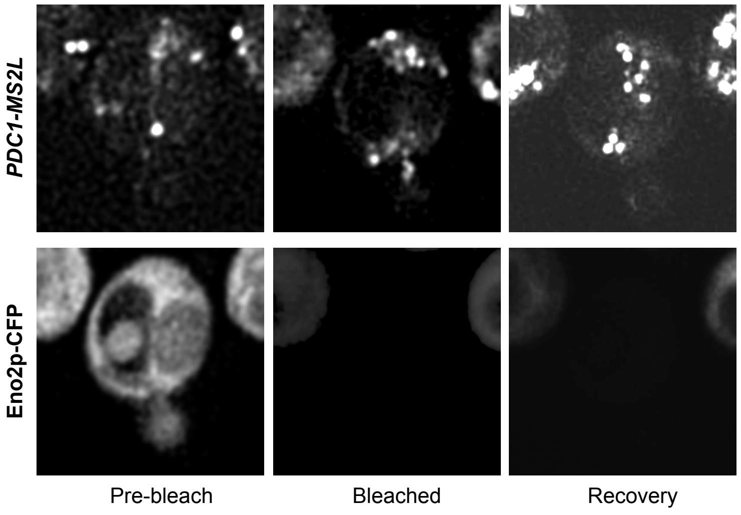

Figure S5. Related to Figure 7. The recovery in flourescence for Eno2p-OFP is negated by cycloheximide treatement. Figure shows a control FRAP experiment for the yMK1993 strain bearing Eno2p-mOrange and the *PDC1-MS2L* mRNA (visualised using MS2-GFP_3). The strain was treated with 100μg/ml cycloheximide prior to bleaching. Pre-bleach, Bleached and Recovery images are shown for *PDC1* mRNA (top row) and mOrange tagged Eno2p protein (bottom row).

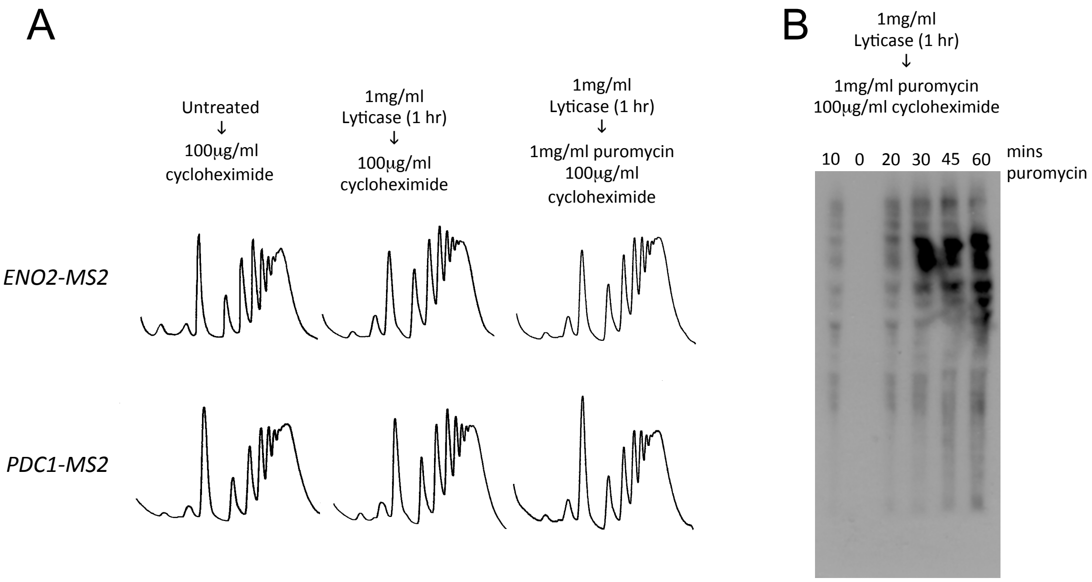

Figure S6. Related to Figure 7. Lyticase treatment allows puromycin to enter cells and under conditions where proteins are puromycylated, cycloheximide prevents puromycin dependent run-off. A. Polysome profiles for the *ENO2-MS2* and *PDC1-MS2* strains following lyticase and puromycin/ cycloheximide treatements as labelled. B. Anti-puromycin antibody probed western blot on whole cell extracts generated after lyticase/ puromycin/ cycloheximide treatment as labelled. Critically in the absence of puromycin no background is detected (lane 0), whereas after as little as 10 minutes puromycin, labelled proteins are observed.
